# α-Hemolysin from *Staphylococcus aureus* Changes the Epigenetic Landscape of Th17 Cells

**DOI:** 10.4049/immunohorizons.2400061

**Published:** 2024-09-06

**Authors:** Joanna Pastwińska, Iwona Karwaciak, Kaja Karaś, Anna Sałkowska, Katarzyna Chałaśkiewicz, Dominik Strapagiel, Marta Sobalska-Kwapis, Jarosław Dastych, Marcin Ratajewski

**Affiliations:** * Laboratory of Epigenetics, Institute of Medical Biology, Polish Academy of Sciences, Lodz, Poland; † Biobank Lab, Department of Oncobiology and Epigenetics, Faculty of Biology and Environmental Protection, University of Lodz, Lodz, Poland; ‡ Laboratory of Cellular Immunology, Institute of Medical Biology, Polish Academy of Sciences, Lodz, Poland

## Abstract

The human body harbors a substantial population of bacteria, which may outnumber host cells. Thus, there are multiple interactions between both cell types. Given the common presence of *Staphylococcus aureus* in the human body and the role of Th17 cells in controlling this pathogen on mucous membranes, we sought to investigate the effect of α-hemolysin, which is produced by this bacterium, on differentiating Th17 cells. RNA sequencing analysis revealed that α-hemolysin influences the expression of signature genes for Th17 cells as well as genes involved in epigenetic regulation. We observed alterations in various histone marks and genome methylation levels via whole-genome bisulfite sequencing. Our findings underscore how bacterial proteins can significantly influence the transcriptome, epigenome, and phenotype of human Th17 cells, highlighting the intricate and complex nature of the interaction between immune cells and the microbiota.

## Introduction

Th17 lymphocytes exhibit a distinctive phenotype, notably, increased levels of the retinoic acid-related orphan receptor RORγt transcription factor and ILs such as IL-17A, IL-17F, IL-21, and IL-22 (1, 2). This subtype of T cells plays a critical role in safeguarding the host against specific pathogens, including *Bacillus anthracis*, *Staphylococcus aureus*, and *Candida albicans* (3–5). Th17 cells have also been implicated in the development of certain autoimmune disorders, such as multiple sclerosis, rheumatoid arthritis, and psoriasis (6–8).

The human body hosts numerous dynamically evolving communities of microorganisms, collectively referred to as the microbiome. These microorganisms form distinct local communities known as microbiota, which can be identified within various bodily regions, such as the oral cavity, nasal cavity, ears, vagina, intestines, lungs, and skin (9). A growing body of evidence suggests that the microbiome plays a pivotal role in maintaining the overall balance within an organism. Alterations in the makeup of the microbiome, referred to as dysbiosis, have the potential to trigger persistent inflammation, a fundamental factor in the pathogenesis of inflammatory bowel disease, diabetes, psoriasis, allergic asthma (10–12), and certain autoimmune disorders, such as rheumatoid arthritis (13, 14). Furthermore, disturbances in the microbiota balance result in immune system dysregulation, which is primarily attributed to the aberrant release of immune system components such as T lymphocytes and cytokines (15, 16). Because *S. aureus* is a common inhabitant of the skin and nasopharyngeal cavity (17) and is a natural pathogen that induces a Th17 response, we investigated how its toxin, α-hemolysin, which is a well-characterized virulence factor of this bacterium (18), affects the transcriptome of Th17 cells. Analysis of the transcriptomes of CD4^+^ cells that differentiated into Th17 lymphocytes in the presence of α-hemolysin revealed a substantial number of genes whose expression was altered. Among these genes were some that were found to be part of the regulatory network for Th17 cells (19) and epigenetic regulators, for example, *HDAC9* (histone deacetylase 9), *DNMT3B* (DNA methyltransferase 3 beta), *CHAF1A* (chromatin assembly factor 1, subunit A [P150]), *HELLS* (helicase, lymphoid specific), *TOP2A* (DNA topoisomerase II alpha), *KDM7A* (lysine demethylase 7A), and *PWWP2B* (PWWP domain containing 2B) that modulate epigenetic processes in cells. Additionally, we observed a marked increase in histone acetylation and other activating or repressive histone marks and changes in global methylation, indicating that this bacterial protein can influence the epigenome of human cells and may induce memory responses or allow the pathogen to evade host defense.

## Materials and Methods

### Reagents

Active, recombinant *S. aureus* α-hemolysin (ab233724) was purchased from Abcam (Cambridge, UK).

### Cell viability

The impact of α-hemolysin on the viability of CD4^+^ cells undergoing differentiation toward Th17 cells was assessed via the CellTiter-Glo luminescent cell viability assay from Promega (Fitchburg, WI) following the manufacturer’s instructions. Luminescence readings for each sample in the 96-well plate were acquired via an Infinite 200 PRO instrument from Tecan (Männedorf, Switzerland).

### Isolation of CD4^+^ cells and Th17 differentiation

The CD4^+^ naive fraction was isolated by utilizing CD4 M-pluriBeads anti-hu (pluriSelect Life Science, Leipzig, Germany) from buffy coats sourced from healthy, anonymous donors. These buffy coats were procured from the Regional Center for Blood Donation and Blood Treatment in Lodz, Poland. The methodology outlined by Wilson et al. (20) was used to induce the differentiation of naive CD4^+^ cells into human Th17 lymphocytes. The cells were cultured in RPMI 1640 medium (PAN-Biotech, Aidenbach, Germany) supplemented with 1% human AB serum and the following cytokines from PeproTech (Rocky Hill, NJ): 50 ng/ml human IL-1β, 30 ng/ml human IL-6, 10 ng/ml human IL-23, and 10 ng/ml human TGF-β. Additionally, beads coated with anti-CD2, anti-CD3, and anti-CD28 Abs (obtained from the T cell activation/expansion kit by Miltenyi Biotec, Bergisch Gladbach, Germany) were added to the culture for a duration of 5 d.

### Preparation of cDNA libraries, RNA sequencing, and data analysis

Primary CD4^+^ cells that were induced to differentiate into Th17 cells over a 5-d period in the presence of 200 ng/ml α-hemolysin were subjected to comprehensive analysis via high-resolution RNA sequencing (RNA-seq), which was performed at Novogene (Cambridge, UK). Initially, total RNA isolation was performed using TRI Reagent from the Molecular Research Center. Subsequently, the isolation of mRNA was carried out using magnetic beads attached to poly(T) oligonucleotides. For the initiation of first-strand cDNA synthesis, random hexamer primers along with Moloney murine leukemia virus reverse transcriptase (RNase H) were used. The subsequent step involved the preparation of second-strand cDNA using DNA polymerase I and RNase H, following a previously described protocol (21). Following an end repair process and 3′ adenylation, adaptors were ligated to the cDNA fragments. Purification of library fragments was conducted using the AMPure XP system (Beckman Coulter, Brea, CA). Subsequent PCR amplification was performed using Phusion high-fidelity DNA polymerase, universal PCR primers, and Index (X) Primer from NEB (Ipswich, MA, USA). The resulting PCR products were purified using the AMPure XP system, and library quality was assessed using the Agilent Bioanalyzer 2100 system (Agilent, Santa Clara, CA). For cluster generation, the cBot cluster generation system along with the TruSeq PE cluster kit v3-cBot-HS from Illumina (San Diego, CA) was used. The subsequent sequencing process was carried out on a NovaSeq 6000 platform (Illumina) using paired-end reads of 150 bp, consistent with previous descriptions (21–23). The initial assessment of the raw read quality was performed using Fast QC software (available at: http://www.bioinformatics.babraham.ac.uk/projects/fastqc). Subsequent filtering and trimming were carried out using bbduk2 v37.10 (accessible at: https://jgi.doe.gov/data-and-tools/bbtools/bb-tools-user-guide/bbduk-guide/). RNA-seq data that successfully passed quality control were then aligned to the human transcriptome utilizing an annotation file (GRCh38) sourced from ENSEMBL. For human rRNA mapping and depletion, Bowtie2 v2.2.6 (24) was used, while for quantification purposes, Salmon software v0.8.2 (25) was used. DESeq2 v1.20.0 (26) was used for differential expression analysis with a fold change cutoff value of 1.5. The gplots package (v3.1.3) in R (https://github.com/talgalili/gplots) was used to generate plots (heatmaps and volcano plots). The RNA-seq results are available under BioProject accession number PRJNA1081854 at the NCBI Short Read Archive (SRA).

### Gene Ontology enrichment analysis and Kyoto Encyclopedia of Genes and Genomes pathway enrichment analysis

Gene Ontology functional annotation and Kyoto Encyclopedia of Genes and Genomes (KEGG) pathway enrichment analysis of the differentially expressed genes (DEGs) were performed with ShinyGO 0.77 (accessible at http://bioinformatics.sdstate.edu/go/) (27, 28). The significance threshold was set at an adjusted *p* value (false discovery rate) of 0.05 for these analyses. In addition, for gene set enrichment analysis, the online tool gsea v3.0, which is based on the KEGG dataset, was used (available at: https://www.gsea-msigdb.org/gsea/index.jsp) (29).

### Whole-genome bisulfite sequencing

Whole-genome bisulfite sequencing (WGBS) was performed using DNA originating from CD4^+^ cells that were isolated and differentiated toward Th17 lymphocytes in the presence of 200 ng/ml α-hemolysin from five different human donors at Novogene (Cambridge, UK). Initially, genomic DNA spiked with λ DNA underwent fragmentation to sizes ranging from 200 to 400 bp. These fragments then underwent bisulfite treatment to generate ssDNA. Subsequently, methylation sequencing adapters were ligated, followed by dsDNA synthesis to construct the library using the Accel-NGS methyl-seq DNA library kit for Illumina (Swift Biosciences, Ann Arbor, MI). Size selection and PCR amplification were performed to prepare the library, which was then quantified using a Qubit system and real-time PCR, and its size distribution was assessed using an Agilent Bioanalyzer 2100 (Agilent, Santa Clara, CA). The quantified libraries underwent pooling and sequencing via Illumina platforms. Following sequencing, the raw reads were subjected to basic statistical analysis using FastQC (fastqc_v0.11.5) to assess quality. The reads generated by the Illumina pipeline in the FASTQ format were subsequently preprocessed with Trimmomatic (version Trimmomatic-0.36) (30). Trimmomatic parameters included SLIDINGWINDOW: 4:15, LEADING: 3, TRAILING: 3, ILLUMINACLIP: adapter.fa: 2:30:10, and MINLEN: 36. Reads that passed all filtering steps were considered clean reads, forming the basis for subsequent analyses. Finally, another round of basic statistical analysis using FastQC was performed on the quality of the clean data reads. Bismark software (version 0.16.3) (31) was used to align bisulfite-treated reads to a reference genome. The reference genome was transformed into a bisulfite-converted form, where C is converted to T and G is converted to A, before being indexed with Bowtie2 (24). Similarly, sequence reads were converted into fully bisulfite-converted versions (C-to-T and G-to-A converted) before alignment to corresponding converted genome versions in a directional manner as described previously (32).

Distinct optimal alignments from both the initial top and bottom strand alignment procedures were juxtaposed with the standard genomic sequence to deduce the methylation status of every cytosine position within the read. Reads aligning to the same genome regions were treated as duplicates. The outcomes generated by the methylation extractor (bismark_methylation_extractor, –no_overlap) were converted to the bigWig format to facilitate visualization through the IGV browser (33). In our efforts to pinpoint methylation sites, we viewed the total count of methylated instances, labeled as Mc, as a binomial random variable with a specified methylation rate. To gauge the methylation level across the sequence, we partitioned the data into multiple bins, each spanning a size of 10 kb. The total counts of methylated and unmethylated reads within each window were computed. The methylation level for each window or C site indicates the ratio of methylated Cs as described previously (34). The calculated methylation level was subsequently adjusted with the bisulfite nonconversion rate, as per prior research (35). Differentially methylated regions (DMRs) were detected utilizing DSS software (36–38). Genes linked with DMRs were identified based on their genomic distribution, considering overlap with either the gene body region (spanning from the transcription start site [TSS] to the transcription end site [TES]) or the promoter region (upstream 2 kb from the TSS) of the DMRs. WGBS results are available under BioProject accession number PRJNA1085862 at the NCBI SRA.

### Real-time RT-PCR

Total RNA isolation was carried out using TRI Reagent sourced from the Molecular Research Center (Cincinnati, OH). The resulting RNA was then suspended in nuclease-free water, with 5 µg of the isolated RNA serving as the input for cDNA synthesis via the Maxima first-strand cDNA synthesis kit from Thermo Scientific/Fermentas (Vilnius, Lithuania), following the manufacturer’s protocol. Quantitative real-time PCR (RT-qPCR) was conducted on a LightCycler 480 system (Roche, Basel, Switzerland) with SYBR Green I master mix (Roche). The PCR cycling conditions included initial denaturation at 95°C for 5 min, followed by 40 cycles of denaturation at 95°C for 10 s, annealing at 60°C for 10 s, and elongation at 72°C for 20 s. The primer pairs used for detecting the target genes were taken from previous works for *RORγt*, *IL17A*, (39) and *IL17F* (40). The primers used for the detection of *IL22*, *DNMT3B*, *HDAC9*, *CHAF1A*, *HELLS*, *TOP2A*, *KDM7A*, and *PWWP2B* were newly designed. For normalization of the mRNA levels, a geometric mean approach involving three housekeeping genes was adopted: hypoxanthine phosphoribosyltransferase 1 (*HPRT1*), hydroxymethylbilane synthase (*HMBS*), and ribosomal protein L13A (*RPL13A*). This normalization strategy followed the method outlined by Vandesompele et al. (41). All primers used in this study can be found in Supplemental Table I.

### IL-17 ELISA

To analyze IL-17 production in CD4^+^ T cells cultured in the presence of increasing concentrations of α-hemolysin, the Quantikine human IL-17 immunoassay kit (R&D Systems, Minneapolis, MN) was used according to the manufacturer’s protocol. Following the incubation period, the cells were harvested for RNA isolation, and the supernatants were collected for ELISA. The ELISA measurements were taken at 450 nm using a Sunrise microplate reader (Tecan, Männedorf, Switzerland).

### Western blotting

Primary CD4^+^ cells were seeded on six-well plates at a density of 12 × 10^6^ cells per well and differentiated into Th17 lymphocytes in the presence of 200 ng/ml α-hemolysin for 5 d. The cells were subsequently harvested and lysed as described in detail in a previous study (42). The following Abs were used: anti-CHAF1A (D77D5) (no. 5480), anti-HELLS (no. 7998), anti–topoisomerase IIα (D10G9) (no. 12286), anti-H3K4me (D1A9) (no. 5326), anti-H3K4me2 (C64G9) (no. 9725), anti-H3K4me3 (C42D8) (no. 9751), anti-H3K27me3 (C36B11) (no. 9733), anti-H3K9me (D1P5R) (no 14186), anti-H3K9me3 (D4W1U) (no. 13969), and anti–β-actin (13E5) (no. 4970) (all purchased from Cell Signaling Technology, Danvers, MA); anti-PWWP2B (ab110052), anti-DNMT3B (ab2851), and anti-HDAC9 (EPR5223) (ab109446) (purchased from Abcam, Cambridge, UK); and anti–ac-H3 (06-599) and anti–ac-H4 (06–866) (purchased from Merck, Darmstadt, Germany). HRP-conjugated secondary Ab ab6721 (Abcam) was used for detection, by visualization of specific bands using SuperSignal West Pico chemiluminescent substrate (Thermo Fisher Scientific, Waltham, MA) on a G:Box chemiluminescence imaging system (Syngene, Cambridge, UK).

### Statistical analysis

The data were assessed via Friedman repeated-measures ANOVA on ranks, followed by a Student–Newman–Keuls post hoc test or paired *t* test utilizing SigmaStat v4.0 (Systat Software, San Jose, CA). Significance was determined with a threshold of *p* ≤ 0.05.

### Data availability

RNA-seq results are available under BioProject accession number PRJNA1081854 at the NCBI SRA. WGBS results are available under BioProject accession number PRJNA1085862 at the NCBI SRA.

## Results

**FIGURE 1.**
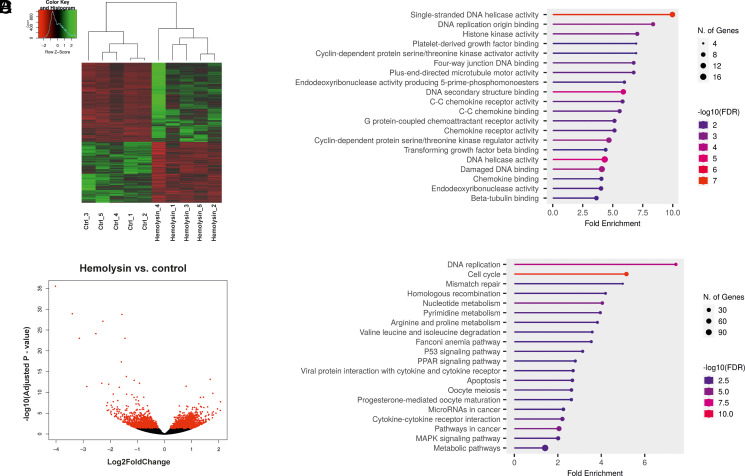
The transcriptome of human Th17 cells undergoing differentiation is influenced by α-hemolysin, as shown by RNA-seq analysis. (**A**) Hierarchical clustering map illustrating the differential gene expression patterns across all samples. Each column represents an individual sample (either a control sample or a sample treated with α-hemolysin), and each row represents a specific gene. Genes exhibiting elevated expression levels are represented in red, and those with decreased expression levels are depicted in green. (**B**) Volcano plot highlights the differentially expressed genes (DEGs) identified upon treatment of CD4^+^ cells undergoing Th17 differentiation with 200 ng/ml α-hemolysin. The red dots represent genes with significantly altered expression, while the black dots indicate genes with nonsignificant changes. (**C**) Results of the molecular function terms of Gene Ontology analysis highlighting the significance of DEGs following α-hemolysin treatment in differentiating Th17 lymphocytes. (**D**) KEGG pathway analysis illustrates the pathways associated with the DEGs following α-hemolysin treatment in differentiating Th17 lymphocytes.

**FIGURE 2.**
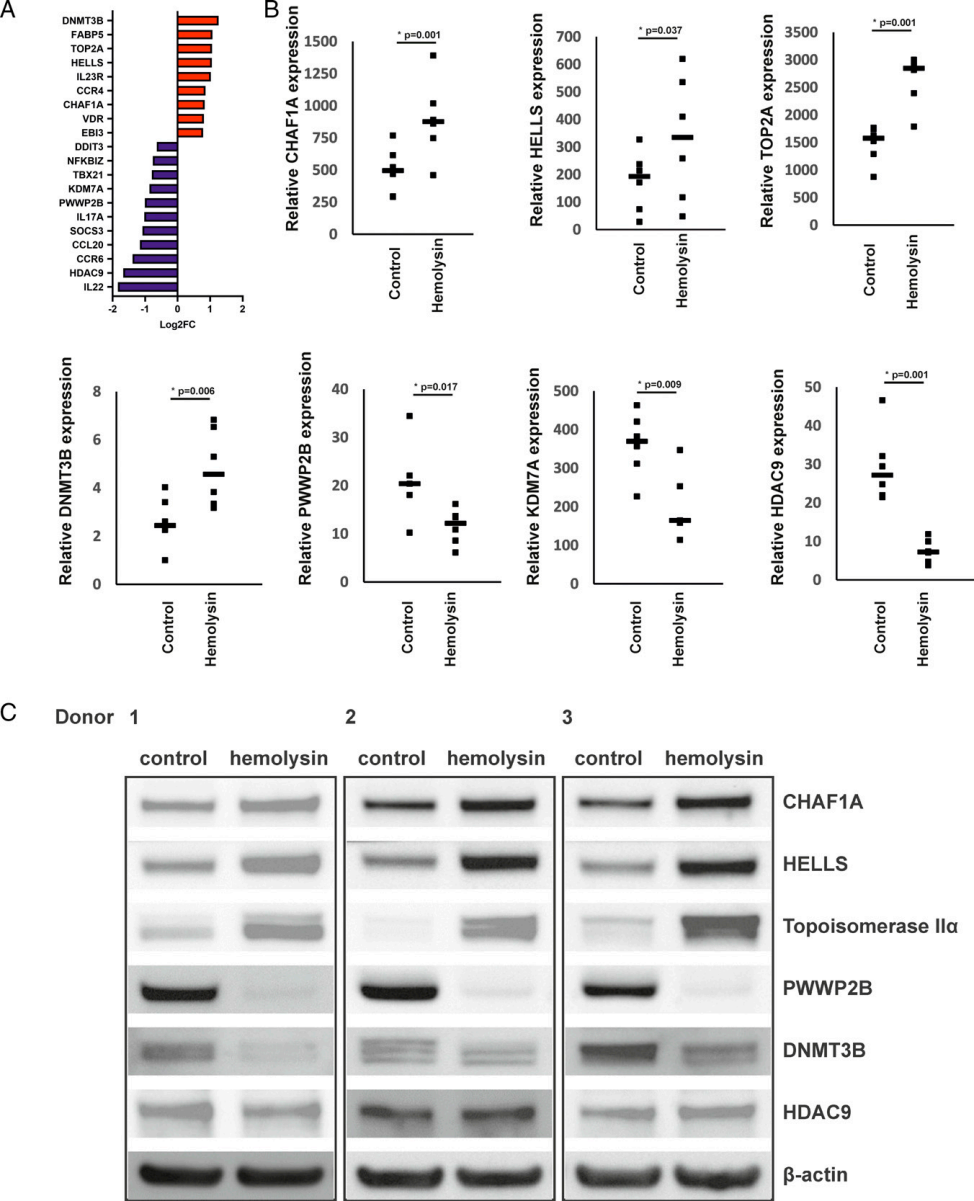
Hemolysin influences genes within the Th17 network and impacts epigenetic regulation. (**A**) Compilation of genes crucial for Th17 cell function and epigenetic regulation identified through a literature review. These genes presented altered expression in differentiating Th17 cells following α-hemolysin treatment, as determined via RNA-seq analysis of data from five (*n* = 5) independent donors. (**B**) Effect of α-hemolysin on the expression of selected genes in differentiating Th17 lymphocytes was examined. CD4^+^ cells isolated from the buffy coats of anonymous donors were differentiated in the presence of α-hemolysin (200 ng/ml) for 5 d. The cells were subsequently harvested, lysed, and subjected to RNA isolation. Real-time quantitative PCR was used to determine the expression levels of *CHAF1A*, *HELLS*, *TOP2A*, *DNMT3B*, *PWWP2B*, *KDM7A*, and *HDAC9*. The results, depicted as a dot plot with median values, were derived from six (*n* = 6) different donors, with statistical significance indicated. *p < 0.05. (**C**) Hemolysin influences the expression of specific proteins involved in epigenetic regulation. CD4^+^ cells obtained from buffy coats of anonymous donors were cultured in the presence of α-hemolysin (200 ng/ml) for 5 d to induce differentiation. The cells were subsequently harvested, lysed, and subjected to Western blotting. The expression levels of CHAF1A, HELLS, topoisomerase IIα, DNMT3B, PWWP2B, HDAC9, and β-actin were assessed via Western blotting. The results depict three blots obtained from three (*n* = 3) different donors.

Th17 cells play crucial roles in defending the human body against *S. aureus* (4, 43). Previous studies have indicated that certain proteins produced by *S. aureus* can elicit an immune response from CD4^+^ lymphocytes (44–47). Therefore, we aimed to investigate the effect of α-hemolysin on the differentiation of human CD4^+^ cells into Th17 lymphocytes. In the initial phase of our study, we investigated how α-hemolysin influences the viability of CD4^+^ lymphocytes undergoing differentiation into Th17 cells. As shown in Supplemental Fig. 1, concentrations up to 200 ng/ml did not induce visible cytotoxicity, confirming that Th17 cells are resistant to this protein (48). We subsequently investigated the effects of this protein on the expression of Th17 signature genes, namely, *RORγt*, *IL17A*, *IL17F*, and *IL22*. We observed significant inhibition of *IL17A*, *IL17F*, and *IL22* expression and secretion of IL-17 in a dose-dependent manner, with the strongest effects occurring at 200 ng/ml α-hemolysin (Supplemental Fig. 2). These findings prompted us to explore how α-hemolysin influences the transcriptome of Th17 cells. To accomplish this goal, we individually differentiated Th17 lymphocytes from CD4^+^ cells isolated from the buffy coats of five healthy donors in the presence of 200 ng/ml α-hemolysin for 5 d and performed RNA-seq. This analysis revealed significant changes in the expression of 1626 transcripts in the cells treated with α-hemolysin (Fig. 1A, 1B, Supplemental Table II). Gene Ontology analysis revealed numerous Gene Ontology terms associated with the regulation of DNA replication, histone kinase activity, DNA secondary structure binding, DNA helicase activity, damaged DNA binding, chemokine receptor activity, chemokine binding, and C-C cytokine binding (Fig. 1C). Pathway enrichment analysis of the DEGs revealed alterations in pathways related to DNA replication, cell cycle regulation, mismatch repair, nucleotide metabolism, apoptosis, cytokine–cytokine receptor interaction, MAPK signaling, and metabolic pathways in cells treated with hemolysin (Fig. 1D), and these results might be consistent with reduced cell proliferation. A detailed analysis of the DEGs revealed that treatment with α-hemolysin impacted many genes known to be part of the regulatory network for Th17 cells (19), including *CCL20*, *CCR4*, *CCR6*, *DDIT3* (DNA damage inducible transcript 3), *EBI3* (DNA methyltransferase 3 beta), *FABP5* (fatty acid-binding protein 5) *IL17A*, *IL22*, *IL23R*, *NFKBIZ* (NFKB inhibitor zeta), *SOCS3* (suppressor of cytokine signaling 3), *TBX21* (T-box transcription factor 21), and *VDR* (vitamin D receptor) (Fig. 2A). Additionally, among the DEGs, genes involved in epigenetic regulation included *HDAC9*, *DNMT3B*, *HELLS*, *CHAF1A*, *KDM7A*, *PWWP2B*, and *TOP2A*. The expression of the mRNA and protein products of these genes was assessed via quantitative PCR and Western blotting (Fig. 2BC). Consistent with the DEGs, we observed the induction of HELLS, topoisomerase IIa, and CHAF1A, as well as the inhibition of PWWP2B (Fig. 2B, 2C, Supplemental Fig. 5). Unexpectedly, although DEGs and quantitative PCR indicated induction, we observed inhibition of the DNMT3B protein, suggesting that α-hemolysin might also influence protein stability. Regarding HDAC9, the effect on protein levels was not as evident as that observed in the RNA-seq and quantitative PCR results (Fig. 2C, Supplemental Fig. 5).

**FIGURE 3.**
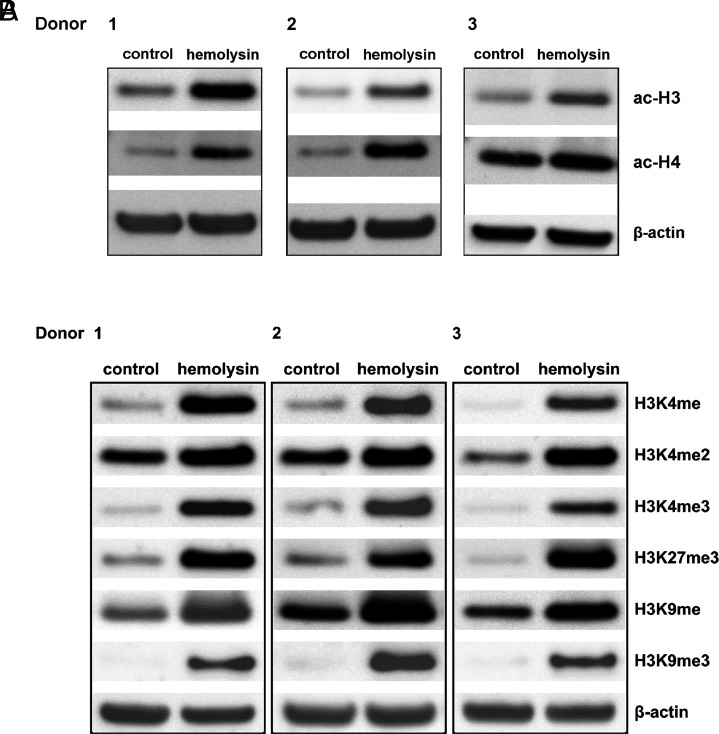
Hemolysin alters the levels of histone marks in human Th17 cells. (**A**) The abundance of acetyl-H3, acetyl-H4, and β-actin were assessed via Western blotting. The results display three blots obtained from three (*n* = 3) different donors. (**B**) The abundances of H4Kme, H4Kme2, H4Kme3, H3K27me3, H3K9me, H3K9me3, and β-actin were analyzed via Western blotting. The results represent three blots obtained from three (*n* = 3) different donors. CD4^+^ cells were extracted from the buffy coats of anonymous donors and cultured in the presence of α-hemolysin (200 ng/ml) for 5 d to induce differentiation. The cells were subsequently harvested, lysed, and subjected to Western blotting.

**FIGURE 4.**
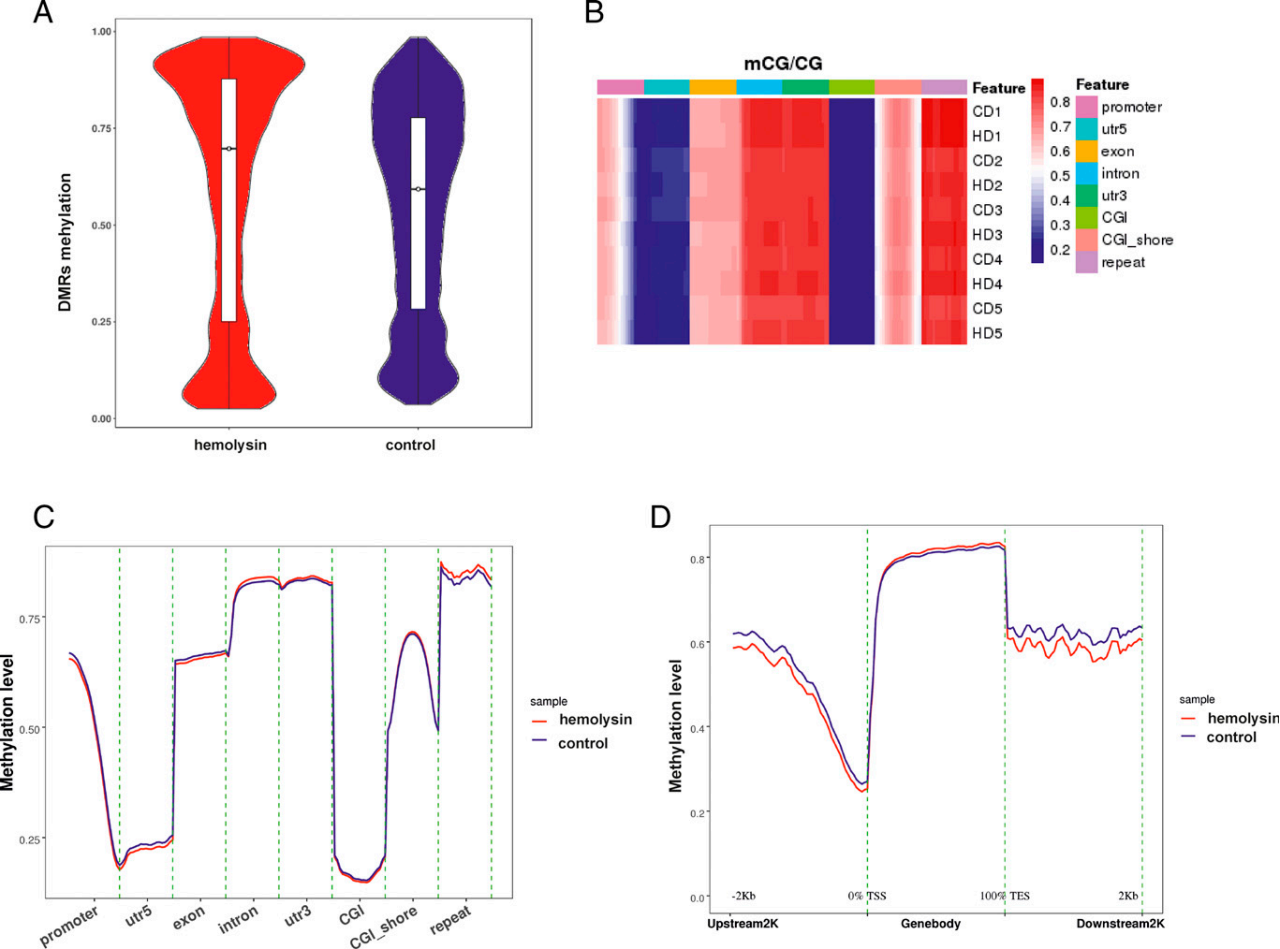
Hemolysin alters the methylation levels of human Th17 lymphocytes, as demonstrated by whole-genome bisulfite sequencing. (**A**) Violin boxplots depicting the comparison between the hemolysin-treated samples and control samples regarding differentially methylated region (DMR) levels of CG. The median value is indicated by the black bar with the empty circle in the middle. (**B**) Heatmap showing the hemolysin-treated samples and the controls depict mCG/CG ratios in the individual samples across various functional genomic regions, as revealed by whole-genome bisulfite sequencing. C, control; D, donor; H, hemolysin. (**C**) Graph comparing the hemolysin-treated samples with the controls depicting the mCG/CG ratios across various functional genomic regions, as revealed by whole-genome bisulfite sequencing. (**D**) Graph showing methylation levels (mCG/CG ratios) across gene bodies and adjacent regions (2 kb upstream and 2 kb downstream). CD4^+^ cells were isolated from the buffy coats of anonymous donors and cultured in the presence of α-hemolysin (200 ng/ml) for 5 d to induce differentiation. The cells were subsequently harvested, lysed, and subjected to DNA isolation, bisulfite treatment, and whole-genome bisulfite sequencing. The data were collected from five different donors.

**FIGURE 5.**
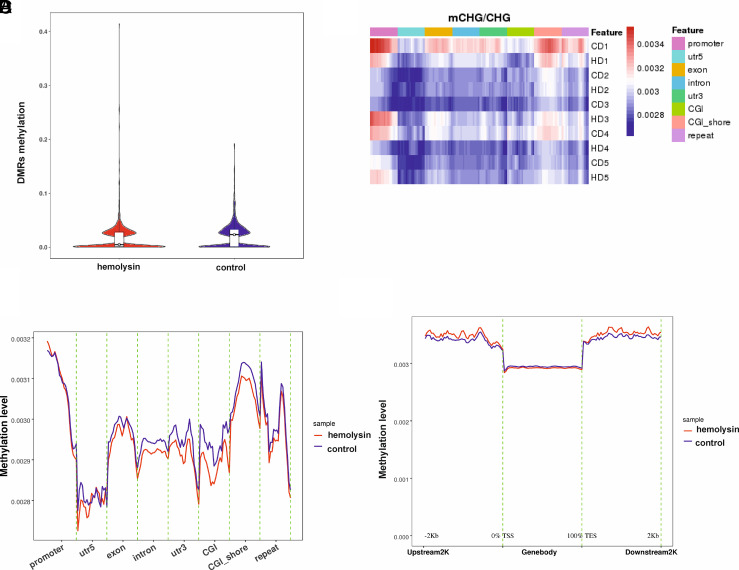
Hemolysin alters the methylation levels of human Th17 lymphocytes, as demonstrated by whole-genome bisulfite sequencing. (**A**) Violin boxplots depicting the comparison between the hemolysin-treated and control samples regarding differentially methylated region (DMR) levels of CG. The median value is marked as a black bar with an empty circle in the middle. (**B**) Heatmap showing the hemolysin-treated samples and the controls depict mCHG/CHG ratios in the individual samples across various functional genomic regions, as revealed by whole-genome bisulfite sequencing. C, control; D, donor; H, hemolysin. (**C**) Graph comparing the hemolysin-treated samples with the controls depicting the mCHG/CHG ratios across various functional genomic regions, as revealed by whole-genome bisulfite sequencing. (**D**) Graph showing methylation levels (mCHG/CHG ratios) across gene bodies and adjacent regions (2 kb upstream and 2 kb downstream). CD4^+^ cells were isolated from the buffy coats of anonymous donors and cultured in the presence of α-hemolysin (200 ng/ml) for 5 d to induce differentiation. The cells were subsequently harvested, lysed, and subjected to DNA isolation, bisulfite treatment, and whole-genome bisulfite sequencing. The data were collected from five different donors.

**FIGURE 6.**
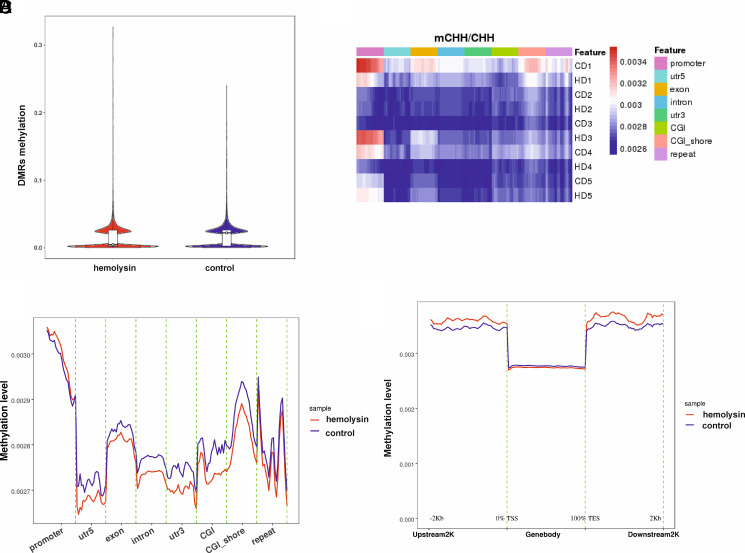
Hemolysin alters the methylation levels of human Th17 lymphocytes, as demonstrated by whole-genome bisulfite sequencing. (**A**) Violin boxplots depicting the comparison between the hemolysin-treated and control samples regarding differentially methylated region (DMR) levels of CHH. The median value is indicated by the black bar with the empty circle in the middle. (**B**) Heatmap showing the hemolysin-treated samples and the controls depict mCHH/CG ratios in the individual samples across various functional genomic regions, as revealed by whole-genome bisulfite sequencing. C, control; D, donor; H, hemolysin. (**C**) Graphs comparing the hemolysin-treated samples with the controls depict mCHH/CHH ratios across various functional genomic regions, as revealed by whole-genome bisulfite sequencing. (**D**) Graph showing methylation levels (mCHH/CHH ratios) across gene bodies and adjacent regions (2 kb upstream and 2 kb downstream). CD4^+^ cells were isolated from the buffy coats of anonymous donors and cultured in the presence of α-hemolysin (200 ng/ml) for 5 d to induce differentiation. The cells were subsequently harvested, lysed, and subjected to DNA isolation, bisulfite treatment, and whole-genome bisulfite sequencing. The data were collected from five different donors.

We next analyzed histone H3 and H4 acetylation. This analysis revealed that α-hemolysin induced histone H3 and H4 acetylation (Fig. 3A, Supplemental Fig. 6). Given that increased CHAF1A expression is associated with other histone marks (49), we investigated whether α-hemolysin affects the abundance of H3K4me, H3K4me2, H3K4me3, H3K27me3, H3K9me, and H3K9me3. Our results indicated that treatment with α-hemolysin induced H3K4me, H3K4me2, and H3K4me3 modifications (Fig. 3B, Supplemental Fig. 7) in Th17 cells, which are generally associated with an active chromatin state (50–52). Moreover, we observed the induction of H3K9me3 and H3K27me3, which are typically considered repressive marks (53, 54). Interestingly, other marks linked with heterochromatin, such as H3K9me (55, 56), were also affected by hemolysin treatment (Fig. 3B, Supplemental Fig. 7). HELLS, either alone or by interacting with methyltransferases, including DNMT3B, is involved in the regulation of transcriptional repression (57–59). Therefore, we investigated the DNA methylation of cell genomes during Th17 differentiation in the presence of α-hemolysin. Cells that differentiated in the presence of α-hemolysin showed different patterns of methylation within chromosomes (Supplemental Fig. 3, Supplemental Table III). As depicted in Fig. 4A, α-hemolysin led to an increase in CG methylation in DMRs and a decrease in CHG and CHH (where H = A, T, or C) methylation (Figs. 5A, 6A). However, this molecule decreased CG methylation in gene promoter regions, 5′-untranslated regions (UTRs), exons, and CpG islands (CGIs) while increasing methylation in introns, 3′-UTRs, and repeated regions (Fig. 4B, C, Supplemental Fig. 4A). With respect to CHG methylation, we observed a decrease in the methylation of 5′-UTRs, exons, introns, 3′-UTRs, CGIs, and CGI shores in the cells treated with α-hemolysin (Fig. 5B, Supplemental Fig. 4B). Hemolysin also resulted in an increase in methylated CHH in promoter regions and a decrease in the methylation of these sites in 5′-UTRs, exons, introns, 3′-UTRs, CGIs, and CGI shores (Fig. 6C, Supplemental Fig. 4C). Analysis of methylation levels in gene bodies and adjacent regions revealed that α-hemolysin decreased the methylation of CGs 2 kb upstream and downstream of gene bodies while increasing methylation within genes (Fig. 4D).

However, the results were different for CHG and CHH. In both cases, α-hemolysin increased methylation in regions upstream and downstream of genes while decreasing methylation levels within gene bodies (Figs. 5D, 6D). We also analyzed the methylation status of promoters and other regions of the DEGs. Among the 1626 DEGs, 66 exhibited changes in promoter methylation status (Table I). Among these DEGs, 48 also presented differences in methylation levels in other regions, such as introns, exons, or UTRs (see Table I). This small number might be somewhat surprising; however, one should consider that α-hemolysin impacts many signaling pathways in cells, and not all changes it causes can be attributed to DNA methylation.

**Table I.** DEGs with hypermethylated and hypomethylated promoters after α-hemolysin treatment

DEG	Promoter/Type	Other Regions/Type
HMGB3	Hypermethylation/CG	Hypermethylation/intron, exon, 5′-UTR, intron/CG
PDZD4	Hypermethylation/CG Hypomethylation/CG	Hypermethylation/exon, intron/CG Hypomethylation/exon, 5′-UTR, TSS/CG
SPEG	Hypermethylation/CG	Hypermethylation/intron/CG
ATP1B3	Hypermethylation/CG	
CENPI	Hypermethylation/CG	
ADAP1	Hypermethylation/CG	Hypermethylation/intron/CG
SMC4	Hypermethylation/CHH	Hypermethylation/intron, 5′-UTR, exon/CHH
HELLS	Hypermethylation/CHH	
CD70	Hypermethylation/CG	Hypermethylation/exon, 5′-UTR, intron/CHH
PLEKHG3	Hypermethylation/CG	Hypermethylation/intron/CG
TNNT3	Hypermethylation/CG	Hypermethylation/intron/CG
PTGFRN	Hypermethylation/CHH	Hypermethylation/intron/CHH Hypomethylation/intron/CHH
KCTD3	Hypermethylation/CHH	Hypermethylation/intron/CHH
ZGRF1	Hypermethylation/CHH	Hypermethylation/intron/CHH
SLC16A2	Hypermethylation/CG	
DIP2C	Hypermethylation/CG	Hypermethylation/exon, intron/CG Hypomethylation/intron/CHG
TIAM1	Hypermethylation/CHH	Hypermethylation/exon, intron/CHH
ZNF572	Hypermethylation/CHH	
RGS6	Hypermethylation/CG	Hypermethylation/exon, intron/CG
RYR1	Hypermethylation/CG	Hypermethylation/exon, 3′-UTR, intron/CG
MT-CYB	Hypermethylation/CHH	
MT-TT	Hypermethylation/CHH	
LTC4S	Hypermethylation/CG	Hypermethylation/TSS, exon, 5′-UTR/CG
FAAHP1	Hypermethylation/CHH	Hypermethylation/intron/CHH
PRR34-AS1	Hypermethylation/CG	Hypermethylation/intron/CG
ETV5	Hypermethylation/CG	Hypermethylation/intron/CG
ESPNP	Hypermethylation/CG	Hypermethylation/intron/CG
ADAMTS7P1	Hypermethylation/CG	Hypermethylation/intron/CG
CACNG4	Hypomethylation/CG	Hypomethylation/intron/CG
WHRN	Hypomethylation/CHH	Hypomethylation/exon, 5′-UTR, intron/CHH
POLA1	Hypomethylation/CG	Hypomethylation/TSS, 5′-UTR, exon, intron/CG
CCNE1	Hypomethylation/CG	
PRPF40B	Hypomethylation/CG	Hypomethylation/exon, 5′-UTR, intron/CG
CDK2AP1	Hypomethylation/CG	Hypomethylation/intron/CG
PHF1	Hypomethylation/CG	Hypomethylation/intron/CG
ADAM23	Hypomethylation/CG	Hypermethylation/intron/CHH Hypomethylation/5′-UTR, exon/CG
HOXB3	Hypomethylation/CG	Hypermethylation/intron/CG Hypomethylation/intron, intron/CG
PREX1	Hypomethylation/CG	Hypomethylation/intron/CHH
THRA	Hypomethylation/CHH	Hypermethylation/intron/CG
TST	Hypomethylation/CG	Hypomethylation/intron, exon, 5′-UTR/CG
ARHGEF11	Hypomethylation/CG	
DTX1	Hypomethylation/CG	Hypomethylation/exon, 5′-UTR/CG
STXBP1	Hypomethylation/CG	Hypomethylation/exon, 5′-UTR, intron/CG
SHF	Hypomethylation/CG	Hypomethylation/exon, 5′-UTR, intron/CG
PIANP	Hypomethylation/CG	Hypomethylation/intron, exon, 5′-UTR/CG
GALNT1	Hypomethylation/CG	Hypomethylation/intron/CG
KIF2C	Hypomethylation/CG	
IGSF3	Hypomethylation/CHH	
NSDHL	Hypomethylation/CG	
PRDM8	Hypomethylation/CG	Hypomethylation/intron/CG
JAZF1	Hypomethylation/CG	Hypomethylation/5′-UTR, TSS, exon/CG
ZCCHC18	Hypomethylation/CG	Hypomethylation/intron/CG
MSRB3	Hypomethylation/CHH	Hypomethylation/intron/CHH
CD248	Hypomethylation/CHH	
ATG9B	Hypomethylation/CG	Hypomethylation/intron/CG
POU6F1	Hypomethylation/CG	
PRR5	Hypomethylation/CG	Hypomethylation/intron/CG
NHS	Hypomethylation/CHG	Hypermethylation/intron/CHG Hypomethylation/intron/CHG
HMGB1	Hypomethylation/CG	Hypomethylation/intron/CG
NUDT11	Hypomethylation/CG	Hypomethylation/exon, 5′-UTR, TSS/CG
FAM72A	Hypomethylation/CG	
GPRASP1	Hypomethylation/CG	Hypomethylation/intron/CG
DMD	Hypomethylation/CG	Hypermethylation/intron/CG Hypomethylation/intron, exon, 5′-UTR/CHG
CCNL2	Hypomethylation/CHH	
ATP5F1AP1	Hypomethylation/CHG	
SSTR3	Hypomethylation/CG	Hypomethylation/exon, 5′-UTR, TSS/CG

## Discussion

Immune cells are constantly interacting with microbiome components. These interactions impact both sides. The scientific literature extensively documents how specific bacterial products can influence the expression of human genes at the transcriptional and epigenetic levels (60–62). These findings motivated us to investigate the effect of α-hemolysin from *S. aureus* on differentiating human Th17 cells, given the role of these lymphocytes in the immune response against this pathogenic bacterium (4). There are several host proteins that mediate the action of α-hemolysin. One of them is ADAM metallopeptidase domain 10 (ADAM10), to which hemolysin binds (63). Other proteins recently identified via genetic screening include SYS1 (Sys1 Golgi trafficking protein), ARFRP1 (ADP-ribosylation factor 1), TSPAN14 (tetraspanin 14), SGMS1 (sphingomyelin synthase 1), ZNF391 (zinc finger protein 391), and HSPA4L (heat shock protein family A [Hsp70] member 4 like) (64). The expression of only two of these genes changed in the system we analyzed, that is, *ZNF391* and *HSPA4L*. However, notably, we observed that the induction of ADAM metallopeptidase domain 9 (ADAM9), which is considered a promoter of inflammation, was able to cleave ADAM10 (65, 66), potentially protecting Th17 cells from α-hemolysin–mediated toxicity (Supplemental Table II). Previous studies utilizing PBMCs, CD4 T cells, and primary Th17 cell clones derived from patients with atopic dermatitis have demonstrated that α-hemolysin induces IL-17 release (46). However, our results are in striking contrast to the results of Niebuhr et al. (46), as we showed that, when CD4^+^ cells were differentiated in the presence of α-hemolysin (Supplemental Figs. 1, 2) for 5 d under Th17-polarizing conditions, *IL17A*, *IL17F*, and *IL22* expression was inhibited, whereas *RORγt* expression remained unaffected (Supplemental Fig. 2). It seems that LPS, a well-known inducer of the Th17 response (67, 68), is not a factor in this discrepancy, as the authors did not detect LPS in their samples (46). Nevertheless, in this study, IL-17 expression and secretion was not stimulated by α-hemolysin at the concentrations used by Niebuhr et al. We cannot exclude the possibility that these differences are due to the protein itself. This is indicated by the fact that the authors of the cited work observed lytic effects of α-hemolysin at concentrations >1 μg/ml, whereas we observed such effects at concentrations four times lower; however, it is noteworthy that there was also a difference in timepoints between these two studies. Moreover, different strains of *S. aureus* have varying abilities to induce an immune response (69), and α-hemolysin itself shows significant genetic diversity, which affects its pathogenicity (70–73). We believe that this phenomenon is extremely interesting and that further research on how different variants of α-hemolysin affect the response of T cells is warranted.

Transcriptional analysis of CD4^+^ cells undergoing Th17 differentiation in the presence of α-hemolysin revealed significant alterations in the expression of a substantial portion of genes from the Th17 network (Figs. 1, 2). Among the DEGs were genes encoding proteins involved in epigenetic regulation, such as *CHAF1*A, *HELLS*, *TOP2A*, *PWWP2B*, *DNMT3B*, *HDAC9*, and *KDM7A*. Consequently, we delved deeper into this epigenetic pathway. Given that *HDAC9* (74–76), *PWWP2B* (77–79), and *TOP2A* (80) are implicated in or associated with histone acetylation, we examined whether α-hemolysin induces the acetylation of histones H3 and H4. As depicted in Fig. 3A, treatment with α-hemolysin indeed increased the acetylation of histones H3 and H4 in cells cultured in the presence of α-hemolysin. We also examined other histone-activating and repressive marks, as many of the genes analyzed were associated with these marks (49, 81, 82). This analysis revealed that α-hemolysin induces both activating and repressive histone marks (Fig. 3B). This finding underscores the complexity of hemolysin activity, which cannot be definitively attributed to either activation or inhibition of epigenetic events. Previous studies have demonstrated that several bacterial proteins can posttranscriptionally modify host signaling molecules, facilitating bacterial invasion and replication (83, 84). Additionally, among these bacterial effector proteins, those containing SET domains, which can methylate host histones, have attracted particular attention (85). One such protein is NUE (protein nuclear effector) from *Chlamydia trachomatis*, which, during infection, translocates to the nucleus and, in in vitro studies, targets the H2B, H3, and H4 histones (86). Another example is the *Legionella pneumophila* effector protein with a SET domain, namely, RomA. This protein contains a nuclear localization signal and can methylate H3K14, thereby reducing acetylation at this site (87–89). Additionally, Mujtaba et al. (90) identified BaSET (suppressor-of-variegation, enhancer-of-zeste, trithorax protein from B. anthracis) from *Bacillus anthracis*, which methylates histone H1 and suppresses genes involved in the activation of inflammatory reactions mediated by NF-κB signaling. The protein products of *Helicobacter pylori* have been shown to inhibit the acetylation of H3K23 in host cells (91), whereas the *Listeria monocytogenes* protein InlB promotes the translocation of sirtuin 2 to the nucleus, resulting in the deacetylation of H3K18 (92). As α-hemolysin does not contain a SET domain, this protein likely does not directly modulate histones. Instead, α-hemolysin likely induces changes in the methylation and acetylation of histones in Th17 lymphocytes by regulating the expression of genes or protein products involved in the process, similar to what has been demonstrated for proteins from *Anaplasma phagocytophilum* or *Escherichia coli* (93, 94). As DNMT3B, HELLS, and CHAF1A (81, 95–99) are directly or indirectly associated with DNA methylation (e.g., through chromatin assembly), we investigated how the methylation of the genome is affected in cells treated with α-hemolysin. Interestingly, we observed significant changes in genome methylation in cells undergoing differentiation in the presence of α-hemolysin. In general, α-hemolysin caused an increase in CG methylation and a decrease in CHG and CHH methylation in DMRs (Fig. 4, Supplemental Figs. 4–6). The increase in CG methylation was particularly prominent in introns, 3′-UTRs, and repeated regions, the latter of which may be associated with the induced expression of HELLS. HELLS facilitates the methylation of repetitive DNA elements and contributes to genome stability (81, 100, 101) (Fig. 4). However, the decrease in non-CpG methylation (CHG and CHH) may be explained by the decrease in the protein levels of DNMT3B in the cells treated with α-hemolysin (Supplemental Figs. 4, 5) (102). An increase in methylation in repeat regions and introns may be a protective response of the cell to α-hemolysin treatment. This response aims to silence repetitive elements that might become activated under stress conditions, serving as a mechanism to maintain genomic stability by suppressing the activity of these potentially disruptive elements (103, 104). Interestingly, only ∼4% of the DEGs had altered methylation statuses within their promoters and other gene regions (see Table I). However, as methylation or demethylation is currently believed to be associated with processes of differentiation and development rather than with single gene regulation (105, 106), we refrained from linking changes in methylation status directly to gene expression.

In conclusion, this study demonstrated that α-hemolysin can alter the expression of a substantial portion of genes crucial for the function of human Th17 lymphocytes and influence the expression of genes involved in epigenetic regulation. Our findings revealed that this toxin induces changes in histone marks and affects genome methylation, shaping the transcriptome, epigenome, and phenotype of Th17 lymphocytes. Owing to the global nature of these observations, we are unable to correlate specific genes or proteins with unique modifications in histones or DNA. Importantly, however, note that we observed the inhibition of certain Th17-associated genes, such as *IL17A*, *IL22*, and *IL23R*. This finding suggests that α-hemolysin might attenuate the Th17 response, potentially explaining how *S. aureus* can lead to recurrent infections in many patients within a few months after antibacterial treatment (107–111). This hypothesis is consistent with a potential mechanism linking epigenetic alterations in Th17 cells following exposure to *S. aureus* to the inhibition of the proinflammatory potential of these cells. In particular, Th17 lymphocytes are long-lived cells with some characteristics of memory cells (112, 113). This notion is supported by multiple observations that bacteria modulate the host response to increase their chances of replication (48, 84). Furthermore, given the lack of successful Th17-targeted therapeutics for autoimmune disorders (114), it is tempting to explore the potential use of modified α-hemolysin to reduce Th17-mediated inflammation in autoimmune conditions. However, because our work is limited to Th17 cells, future studies are needed to investigate whether α-hemolysin also affects the epigenomes of other T lymphocyte subpopulations or other cell types to influence and shape the immune response to *S. aureus*.

## Supplementary Material

Supplemental Material (PDF)

Supplementary DataSet 1 (XLS)

Supplementary DataSet 2 (XLS)
