# Supplementary material for: α-Hemolysin from Staphylococcus aureus Changes the Epigenetic Landscape of Th17 Cells
Source: Immunohorizons. 2024 Sep 6;8(9):606–21. doi: 10.4049/immunohorizons.2400061 (PMC11447695; doi:10.4049/immunohorizons.2400061)
Supplement: Supplemental Material (PDF) [file IH_2400061_Supplemental_1.pdf]

Supporting information

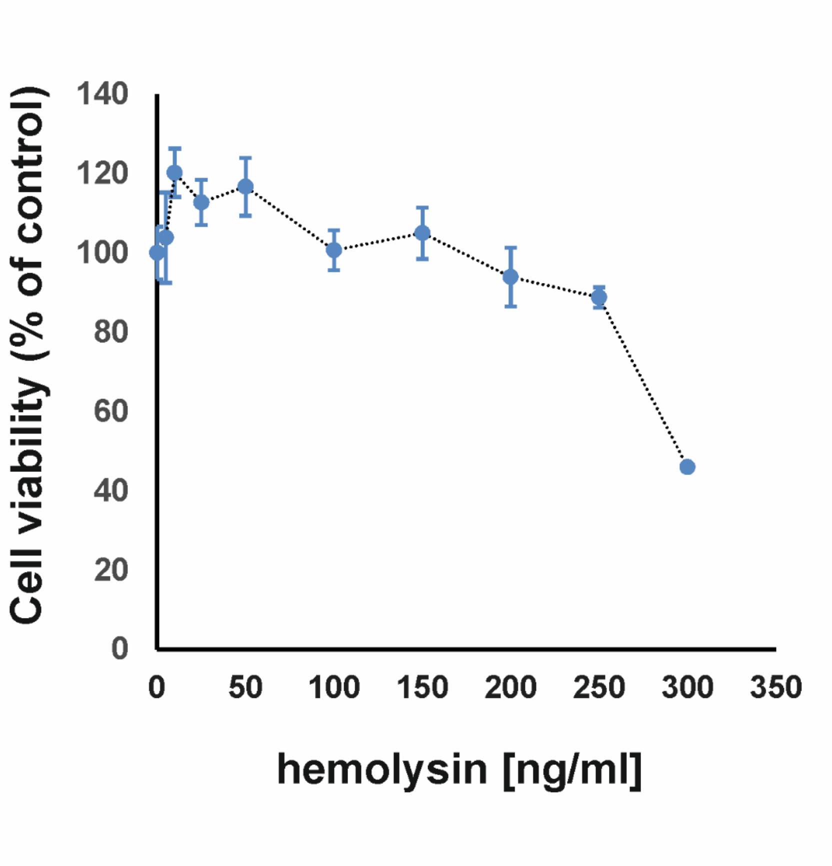

Supplementary Figure 1. Effect of α-hemolysin on differentiating Th17 lymphocytes. Cell viability of naive CD4+ cells differentiated in the presence of increasing concentrations of α-hemolysin for 5 days. The results are shown as the mean ±SD from cells isolated from 3 donors (n=3).

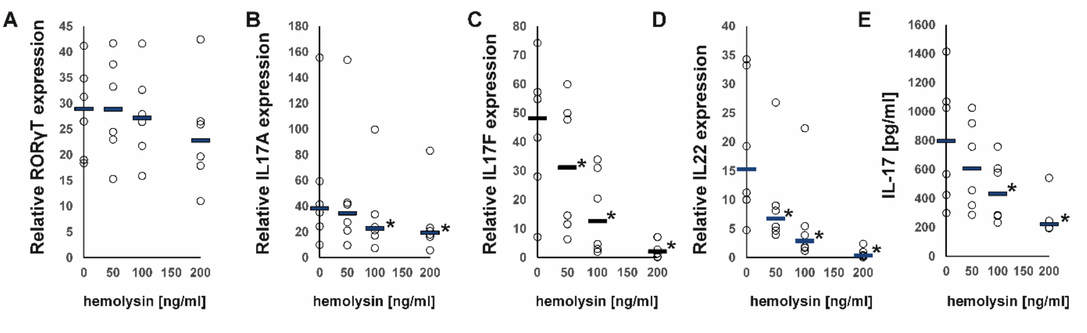

Supplementary Figure 2. The impact of α-hemolysin on specific genes within differentiating Th17 lymphocytes was investigated. CD4+ cells were extracted from the buffy coats of anonymous donors and cultured in the presence of α-hemolysin (200 ng/ml) for 5 days. Following this incubation period, the cells were harvested and lysed, and RNA was isolated. Real-time quantitative PCR was used to assess the expression levels of RORγT, IL17A, IL17F, and IL22. IL-17 ELISA was determined in the supernatants using the Quantikine Human IL-17 Immunoassay Kit. The results, depicted as a dot plot with median values, were derived from six (n=6) different donors. Statistical significance (* $p < 0.05$) is indicated.

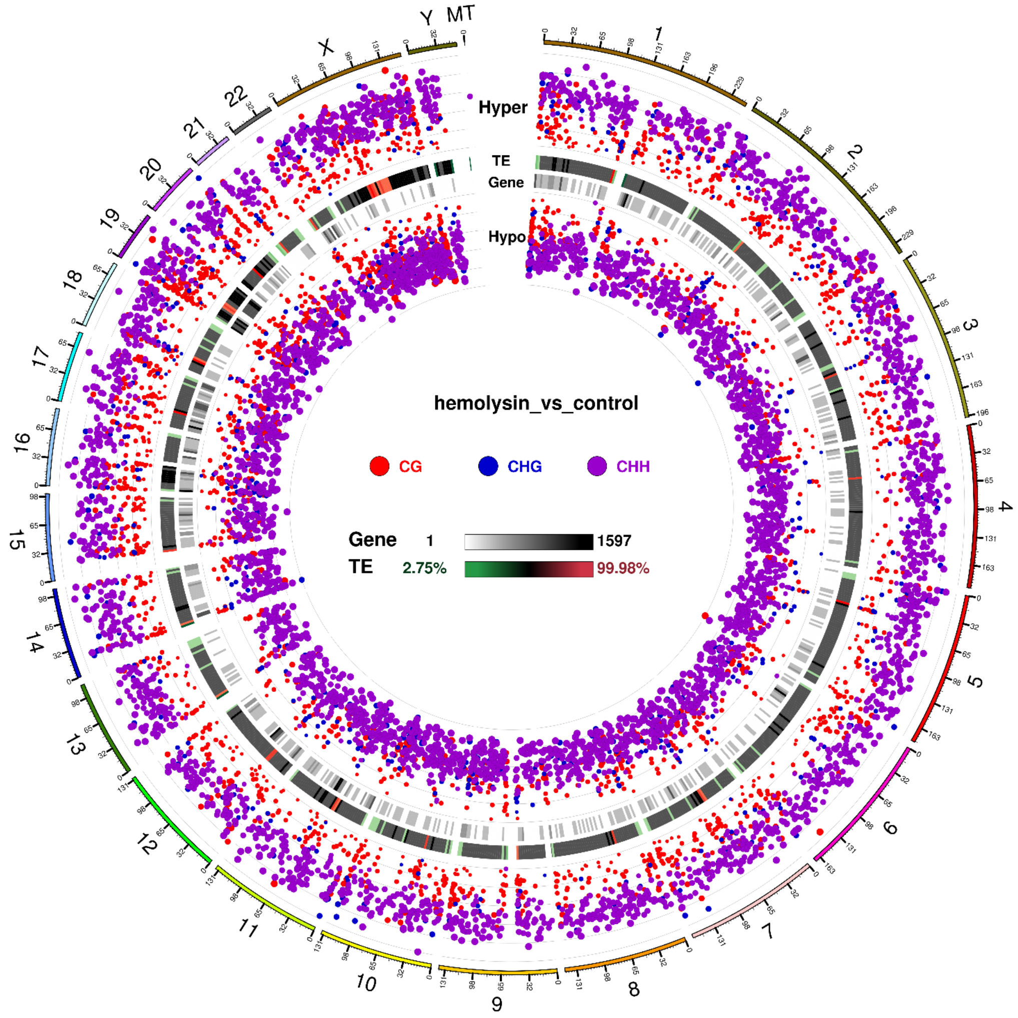

Supplementary Figure 3. The Circos plot showing the significance and distribution of hemolysin-treated vs. control DMRs on specific chromosomes in all contexts (CG/CHG/CHH). The hyper-

DMR and hypo-statistical values were log5(|areaStat|). The higher and larger the point is, the larger the differences between the two groups. The CG context is shown as a red circle, the CHG context is shown as a blue circle, and the CHH context is shown as a purple circle. TE shows the heatmap of the percentage of repeat elements. The genes from 1 to 1597 are shown in a heatmap of gene density.

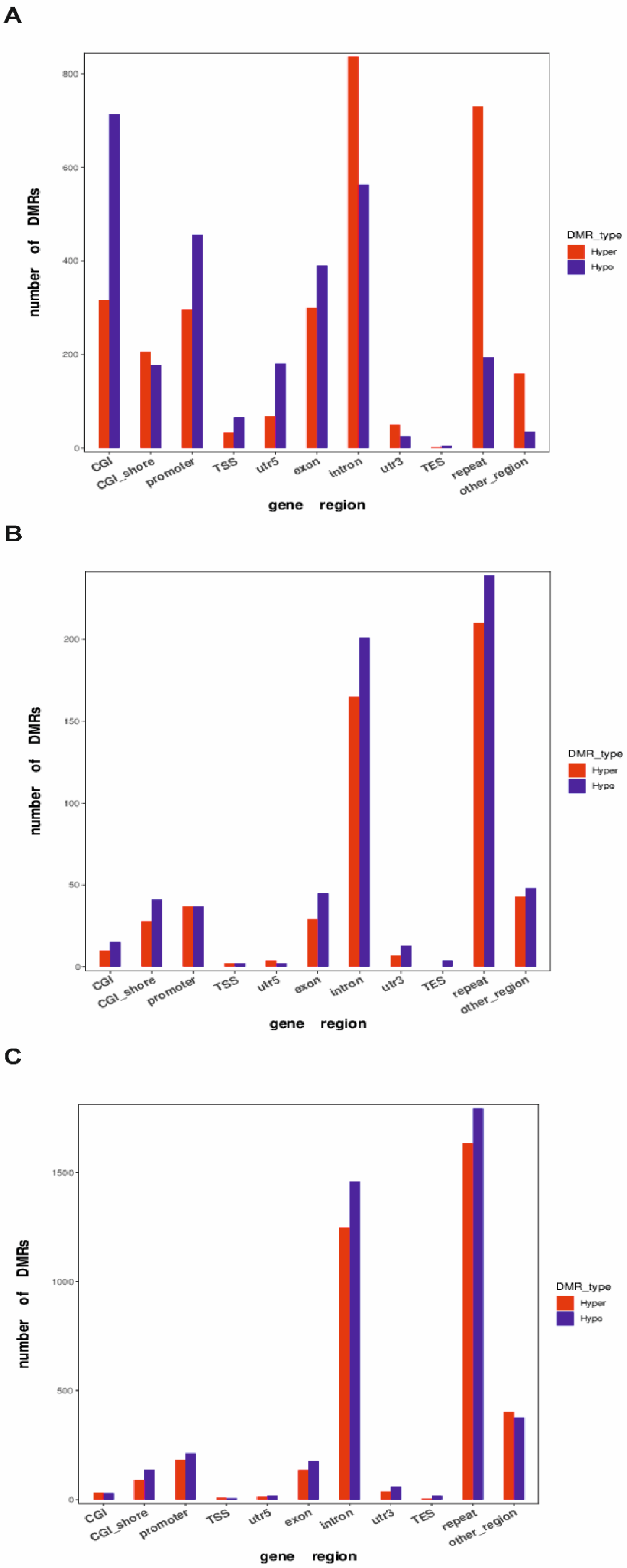
A
number of DMRs
0
200
400
600
800
CGI
CGI_shore
promoter
TSS
utr5
exon
intron
utr3
TES
repeat
other_region
gene region
DMR_type
Hyper
Hypo
B
number of DMRs
0
50
100
150
200
CGI
CGI_shore
promoter
TSS
utr5
exon
intron
utr3
TES
repeat
other_region
gene region
DMR_type
Hyper
Hypo
C
number of DMRs
0
500
1000
1500
CGI
CGI_shore
promoter
TSS
utr5
exon
intron
utr3
TES
repeat
other_region
gene region
DMR_type
Hyper
Hypo

Supplementary Figure 4. DMR gene region distribution of CG (A), CHG (B), and CHH (C) in the hemolysin vs. control groups as demonstrated by whole-genome bisulfite sequencing. CD4+ cells were obtained from buffy coats of anonymous donors and cultured in the presence of α-hemolysin (200 ng/ml) for 5 days to induce differentiation. Subsequently, the cells were harvested, lysed, and subjected to DNA isolation, bisulfite treatment, and whole-genome bisulfite sequencing. The data were obtained from five different donors.

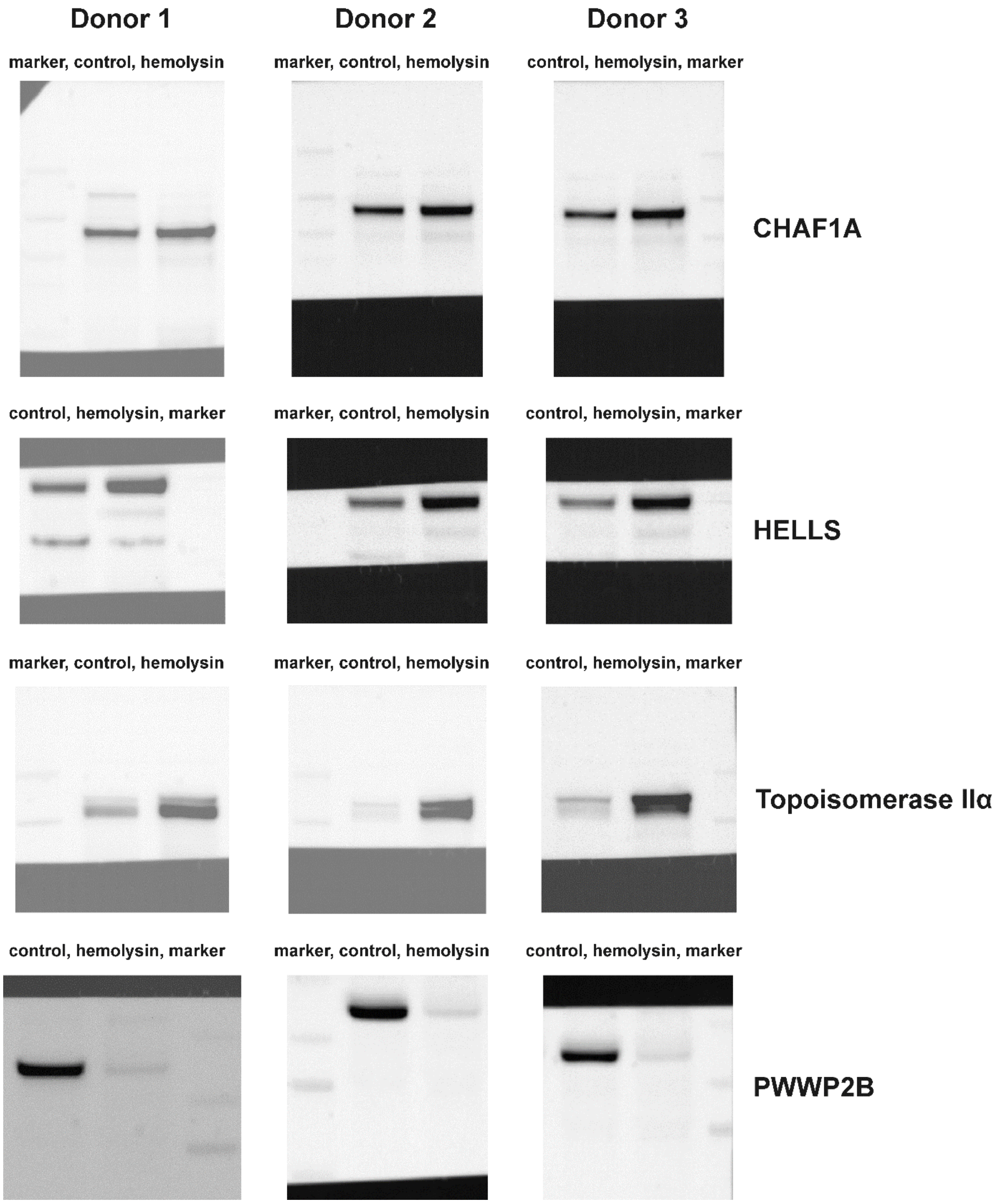

Donor 1
Donor 2
Donor 3
marker, control, hemolysin
marker, control, hemolysin
control, hemolysin, marker
CHAF1A
control, hemolysin, marker
marker, control, hemolysin
control, hemolysin, marker
HELLS
marker, control, hemolysin
marker, control, hemolysin
control, hemolysin, marker
Topoisomerase IIα
control, hemolysin, marker
marker, control, hemolysin
control, hemolysin, marker
PWWP2B

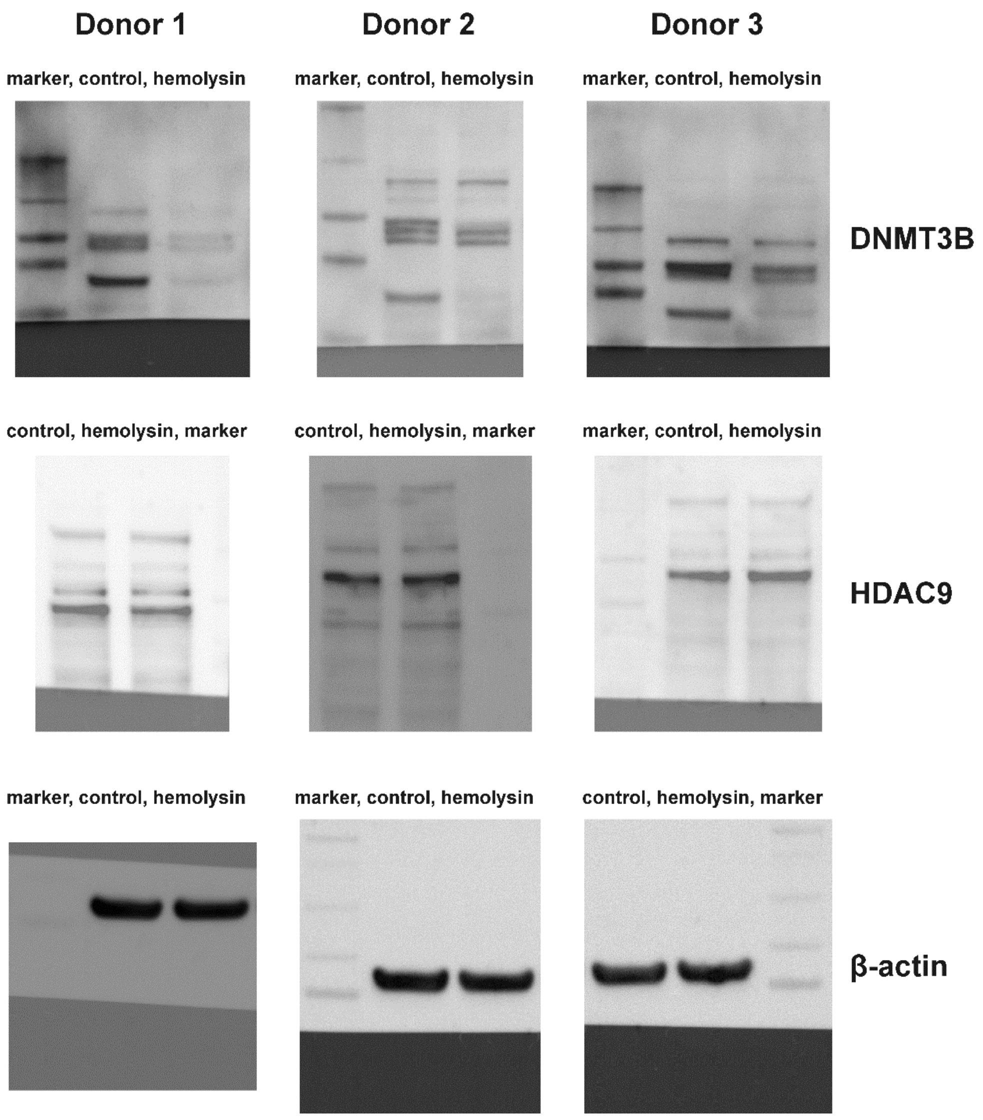

Supplementary Figure 5. Original western blot images for Figure 2.

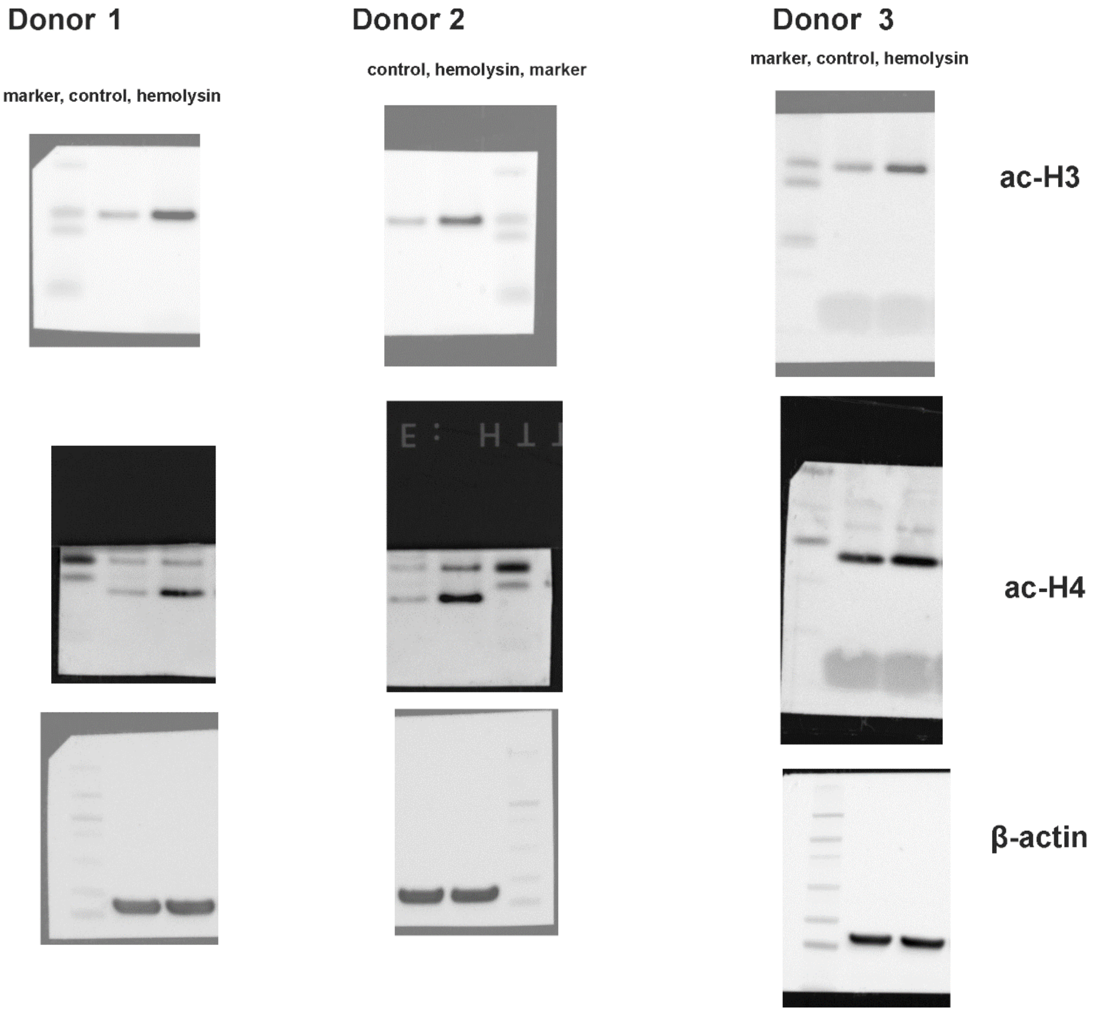

Supplementary Figure 6. Original western blot images for Figure 3A.

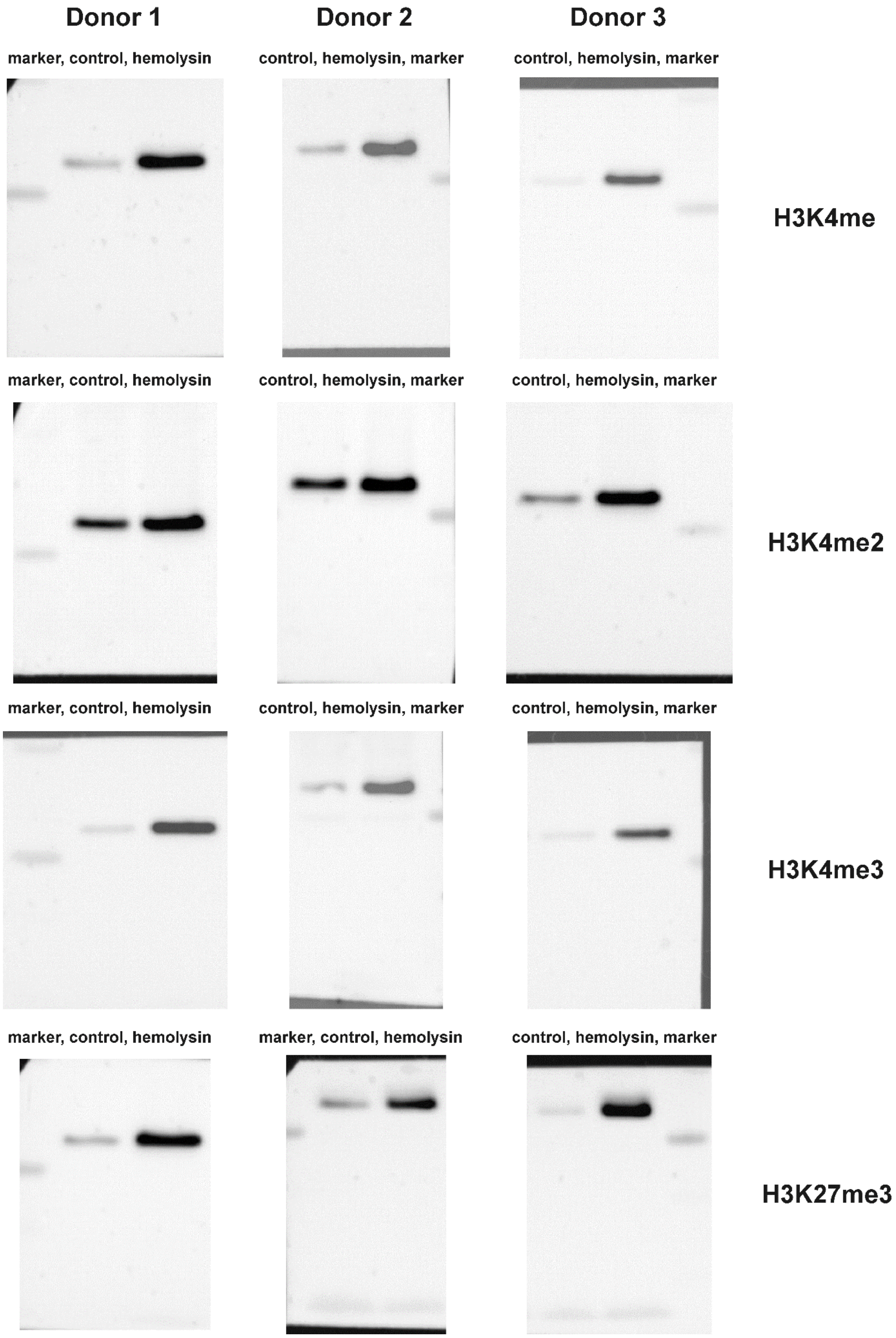
Donor 1
Donor 2
Donor 3
marker, control, hemolysin
control, hemolysin, marker
control, hemolysin, marker
H3K4me
marker, control, hemolysin
control, hemolysin, marker
control, hemolysin, marker
H3K4me2
marker, control, hemolysin
control, hemolysin, marker
control, hemolysin, marker
H3K4me3
marker, control, hemolysin
marker, control, hemolysin
control, hemolysin, marker
H3K27me3

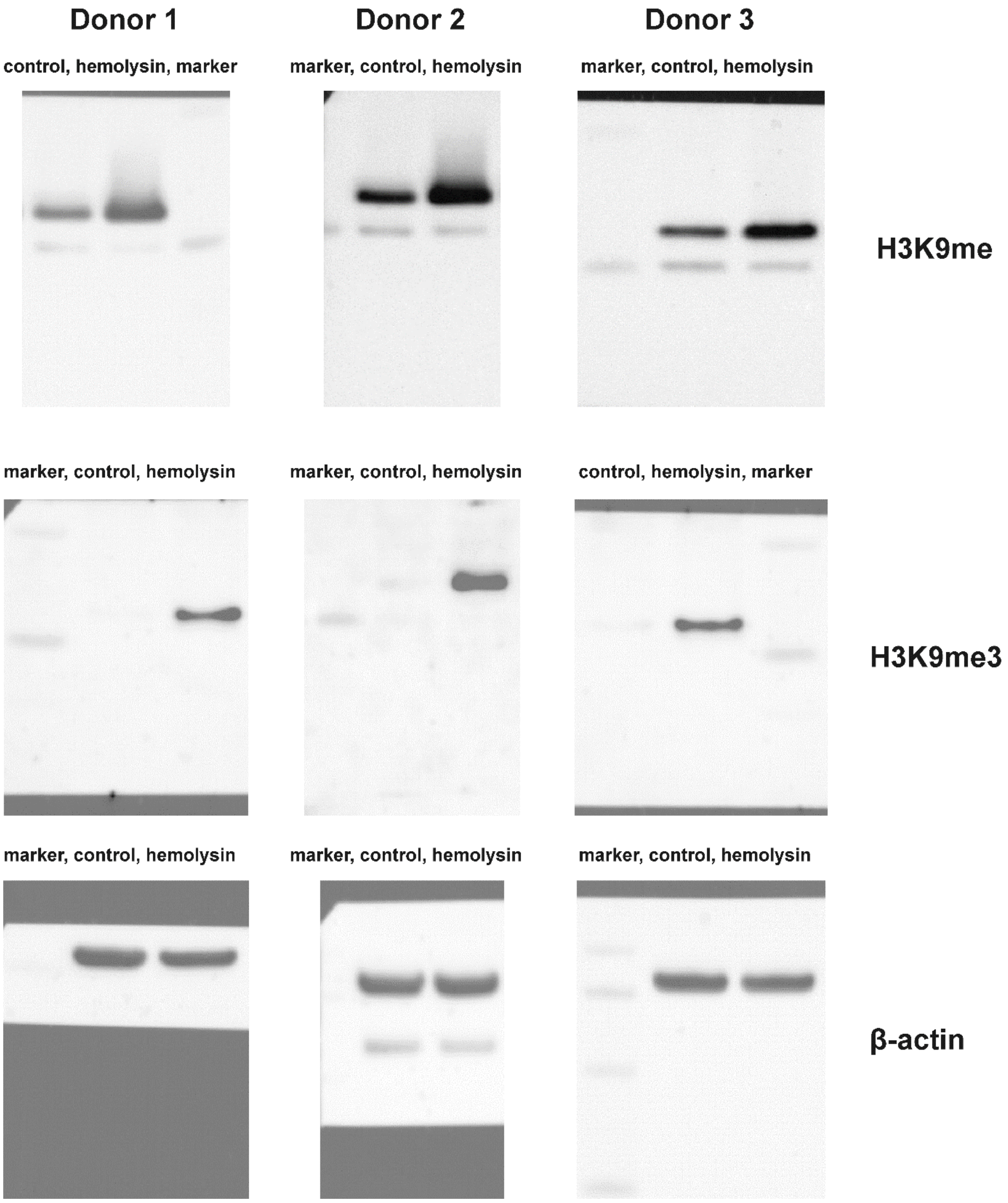

Supplementary Figure 7. Original western blot images for Figure 3B.

Supplementary Table 1. List of primer pairs used in quantitative real time RT-PCR.

Gene	Forward	Reverse
RORγT	5'-CTGCTGAGAAGGACAGGGAG-3'	5'- AGTTCTGCTGACGGGTGC-3'
IL17A	5'-AAACAACGATGACTCCTGGG-3'	5'-CTTGTCCTCAGAATTTGGGC-3'
IL17F	5'-CTTTCTGAGTGAGGCGGC-3'	5'-TGGGAACGGAATTCATGG-3'
IL22	5'- TGGCTGATAACAACACAGACG-3'	5'-GCTTTTGCACATTCCTCTGG-3'
DNMT3B	5'-AGGACTCGTTCAGAAAGCCC-3'	5'-AAGGTAAGAGCTGGGAGGGG-3'
HDAC9	5'-GCATGAGAACTTGACACGGC-3'	5'- TGTTCTCTGCGATGCCTCTC-3'
CHAF1A	5'-CATTCGTCCCTGAGCTCTCC-3'	5'-CTGCACGTAACTTGAGCTGC-3'
HELLS	5'-CTGGAGGAGTGATGCGATGG-3'	5'-GGGCCACAGACAAGAAAAGG-3'
TOP2A	5'-CGCCCAGACACCTACATTGG-3'	5'-TCCCTTTGTTTGTTGTCCGC-3'
KDM7A	5'-ACCGCTTCATGATCGAGTGC-3'	5'-TCTGTGTAGTCATGTCTGTGCC-3'
PWWP2B	5'-GGTGGAGCAGGTCGTCAAC-3'	5'-CCCAGCTGCATCACCTCTG-3'
